# Sputum “IL-5, IL-17A, IL-25-high” pattern is associated with uncontrolled asthma and worse lung function

**DOI:** 10.1186/2045-7022-3-S1-O3

**Published:** 2013-05-03

**Authors:** Sven Seys, Marcin Grabowski, Wim Adriaensen, Ann Decraene, Ellen Dilissen, Jeroen Vanoirbeek, Lieven Dupont, Jan Ceuppens, Dominique Bullens

**Affiliations:** 1 Katholieke Universiteit Leuven, Belgium; 2 Wroclaw Medical University, Department of Physiology, Poland; 3 Katholieke Universiteit Leuven, General Practice, Belgium; 4 Katholieke Universiteit Leuven, Clinical and Experimental Medicine, Belgium; 5 Katholieke Universiteit Leuven, Microbiology and Immunology, Belgium; 6 Katholieke Universiteit Leuven, Public Health, Belgium; 7 University Hospital Leuven, Pneumology, Belgium

## Background

Asthma is a heterogeneous disease with various clinical, inflammatory and molecular phenotypes. Uncontrolled asthma is associated with an increased risk of asthma exacerbation. We evaluated sputum cytokine mRNA expression patterns in an unselected group of adults with stable asthma in comparison to healthy subjects in order to characterize patients’ specific airway cytokine profiles.

## Method

Differential cell counts and cytokine mRNA (quantified by real-time PCR) were analyzed on sputum from 40 healthy and 66 asthmatic adults. A “cytokine-high” profile was defined if mRNA levels for that particular cytokine exceeded the 90th percentile value in the healthy population. Asthma control was assessed by Asthma Control Test (ACT) questionnaires.

## Results

The “IL-5-high” asthma profile (n=13) merged with the “IL-25-high” (10/13) and surprisingly also with the “IL-17A-high” (11/13) profile. A sputum “IL-25-high” profile was even restricted to patients with an “IL-5-high” profile. Only a minority of “IL-5-high” patients were found to be “IL-4-high” (n=6), whereas 17 other patients had an “IL-4-high” profile without being “IL-5-high”. Patients with an “IL-5-high”, Il-17A-high” and/or “IL-25 high” cytokine pattern had worse lung function parameters (FEV1%pred., PEF, FEF25-75%). Uncontrolled asthmatics had significantly higher sputum IL-5, IL-17A and IL-25 mRNA levels.

## Conclusion

Airway cytokine expression is highly heterogeneous amongst patients and their exact contribution to asthma pathogenesis is debated. Uncontrolled asthma was associated with higher levels of IL-5, IL-17A and IL-25 in the airways of asthmatics. Identifying patients’ aberrant cytokine expression in the airways by non-invasive techniques might help to define responders to current and future therapies.

